# Dairy Farm *Streptococcus agalactiae* in a Region of Northeast Brazil: Genetic Diversity, Resistome, and Virulome

**DOI:** 10.3390/pathogens15020128

**Published:** 2026-01-24

**Authors:** Vinicius Pietta Perez, Fernanda Zani Manieri, Luciana Roberta Torini, Carlos Gabriel Andrade Barbosa, Fabio Campioni, Fabiana Caroline Zempulski Volpato, Eloíza Helena Campana, Artur Cezar de Carvalho Fernandes, Afonso Luís Barth, Eduardo Sergio Soares Sousa, Celso Jose Bruno de Oliveira, Ilana Lopes Baratella da Cunha Camargo

**Affiliations:** 1 Departamento de Microbiologia, Imunologia e Parasitologia, Instituto de Ciências Básicas da Saúde, Universidade Federal do Rio Grande do Sul, Ramiro Barcelos Street 2600, Porto Alegre 90035-003, RS, Brazil; 2 Laboratório de Epidemiologia e Microbiologia Moleculares—LEMiMo, Instituto de Física de São Carlos, Universidade de São Paulo, João Dagnone Avenue 1100, São Carlos 13563-120, SP, Brazil; 3 Centro de Ciências da Saúde, Universidade Federal da Paraíba, Campus I. Cidade Universidade, João Pessoa 58051-900, PB, Brazil; 4 Laboratório de Pesquisa em Resistência Bacteriana—LABRESIS, Hospital das Clínicas de Porto Alegre, Universidade Federal do Rio Grande do Sul, Ramiro Barcelos Street 2350, Porto Alegre 90035-903, RS, Brazil; 5 Centro de Ciências Agrárias, Universidade Federal da Paraíba, Campus II. Road PB 079, km 12, Areia 58397-000, PB, Brazil; 6 Centro de Ciências Médicas, Universidade Federal da Paraíba, Campus I. Cidade Universidade, João Pessoa 58051-900, PB, Brazil

**Keywords:** Group B streptococci, GBS, mastitis, cattle, MLST, epidemiology, antibiotic resistance

## Abstract

*Streptococcus agalactiae* is a major cause of bovine mastitis, which affects the quality and yield of milk. The main strategy for controlling this pathogen on dairy farms is the use of antibiotics. This study investigated the clonality, serotype distribution, antimicrobial susceptibility, and presence of resistance and virulence genes in 46 *S. agalactiae* isolates obtained from raw bovine milk in northeastern Brazil. Capsular types were determined using multiplex PCR and antibiotic susceptibility profiles were determined using disc diffusion or the gradient strip method. Clonal diversity was evaluated via pulsed-field gel electrophoresis. Eight isolates were sequenced using short- and long-read methods. There was high overall genetic diversity, whereas the resistance and virulence profiles were largely homogeneous within herds. Tetracycline and macrolide resistance was frequent and mediated by *tetO* and *ermB* and less frequently by *tetM*. Genome analysis demonstrated that resistance genes are present in mobile genetic elements that are also present in human isolates, and phylogenomic analyses identified ST-103 as the predominant and multi-host-adapted lineage, whereas ST-91 clustered with the bovine-adapted lineage. These findings expand the molecular epidemiology of *S. agalactiae* in dairy farms of a region in northeast Brazil and highlight the importance of surveillance strategies for guiding mastitis control and mitigating the spread of antimicrobial resistance.

## 1. Introduction

In 1932, Rebecca Lancefield described two isolates obtained from bovine mastitis milk as Group B Streptococci, *Streptococcus agalactiae* [1]. Since then, this species has been associated with bovine mastitis. In Brazilian dairy herds, *S. agalactiae* remains the main agent of contagious mastitis, with the prevalence varying across regions and according to the characteristics of the dairy production system [2,3]. Contagious mastitis caused by *S. agalactiae* is strongly associated with intensive dairy production systems and the absence or inadequate implementation of mastitis control programs [2,4].

*S. agalactiae* is an intramammary pathogen in cattle, and its introduction into previously negative dairy farms is generally attributed to the acquisition of infected animals from other herds or, more recently, to its ability to survive in environmental sources [5]. Additionally, *S. agalactiae* is a commensal member of the human microbiota and an important cause of perinatal infections, including neonatal meningitis [6]. Some lineages have also been observed in other animals (fish, dolphins, dogs, rats, and frogs), and their zoonotic potential was highlighted in 2015 by an outbreak of bacteremia associated with the consumption of contaminated fish. Thus, *S. agalactiae* epidemiology should be considered within a One Health approach [7].

Several virulence factors contribute to its pathogenicity, such as the polysaccharide capsule playing a key role in immune evasion, fibrinogen-binding proteins (FbsA, FbsB, and FbsC) mediating adhesion to udder cells, hemolysin promoting cellular injury and invasion, and C5a peptidase encoded by *scpB,* recognized as an essential virulence determinant in human-adapted strains [8].

The use of antibiotics remains a primary strategy for controlling *S. agalactiae* in dairy farms. However, the widespread use of antimicrobials in veterinary medicine is a major driver of antimicrobial resistance [9]. Tetracycline (TET) resistance is common in *S. agalactiae*, estimated at 80.1% [10], mainly due to the acquisition of ribosomal protection proteins such as TetM, TetO, and TetS [11]. Additionally, resistance to macrolides, such as erythromycin (ERY), and lincosamides, such as clindamycin (CLI), globally estimated at 30.5% and 29.3%, mediated by 23S rRNA methyltransferases (*erm* genes) or efflux pumps, has emerged as a growing concern in recent years [10]. Resistance to beta-lactams, the first-line therapy, has also been reported in human and bovine mastitis strains [12,13]. These resistance trends highlight the need for the continuous surveillance of antimicrobial susceptibility in *S. agalactiae* to guide effective mastitis control strategies.

Epidemiological and genomic data on *S. agalactiae* in bovine milk from Northeast Brazil are scarce. In this study, we investigated the clonality, serotype distribution, antibiotic susceptibility profiles, presence of antibiotic resistance genes (ARGs), and virulence determinants in *S. agalactiae* isolates obtained from bulk tanks in four municipalities of Paraíba, Brazil. In addition, by integrating whole-genome sequencing (WGS) data generated using both short- and long-read platforms for eight representative isolates, we provide a deeper understanding of the population structure and genomic diversity of circulating *S. agalactiae* lineages.

## 2. Materials and Methods

### 2.1. Bacterial Isolation and Identification

**Figure 1:**
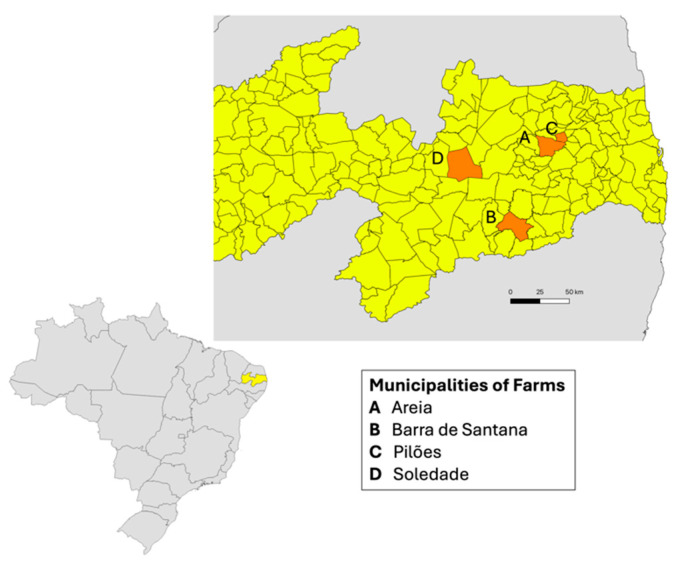
Municipalities in the state of Paraíba, Brazil, where the dairy farms were located, and the 46 *S. agalactiae* isolates were obtained from bovine milk between 2018 and 2022. Including 21 isolates from Areia herd, 23 isolates from Barra de Santana herd, and from Pilões and Soledade one isolate of each herd. The yellow color indicates the state of Paraíba, and the orange color indicates the municipalities included in the study.

This study included 46 *S. agalactiae* isolates that were obtained from raw bovine milk. Samples were collected from four bulk dairy farm tanks in Paraiba State, Brazil (Figure 1), from August 2018 to September 2022. Milk samples were collected under aseptic conditions and transported at 4–8 °C to subsequent microbiological analysis. Samples were inoculated onto blood agar plates supplemented with 5% sheep blood (HiMedia Laboratories, Mumbai, India) and incubated at 35 ± 1 °C for 24–72 h under aerobic conditions. Species-level identification was initially performed using conventional phenotypic methods, including catalase, Christie–Atkins–Munch–Petersen (CAMP), and L-pyrrolidonyl-β-naphthylamide (PYR) tests. Following preliminary identification, isolates were stored in Brain Heart Infusion (BHI) broth supplemented with 10% glycerol at −20 °C. Identification was subsequently confirmed by matrix-assisted laser desorption/ionization time-of-flight (MALDI-TOF) mass spectrometry using the Biotyper 3.1 system (Bruker Daltonics, Bremen, Germany).

### 2.2. Antimicrobial Susceptibility Tests

The isolates were tested for susceptibility to penicillin (PEN), ERY, CLI, chloramphenicol (CHL), levofloxacin (LEV), linezolid (LZD), TET, and vancomycin (VAN) antibiotics using the disc-diffusion method (Cecon, São Paulo, Brazil) on Mueller-Hinton agar supplemented with 5% sheep blood (Newprov, Pinhais, Brazil) according to the Clinical and Laboratory Standard Institute (CLSI) [14]. The PEN minimum inhibitory concentration (MIC) was determined using the gradient strip method (E-test, Biomerieux, Marcy-l’Étoile, France) and evaluated according to the CLSI’s interpretive criteria [14].

### 2.3. Capsular Serotyping

The capsular type of each isolate (Ia, Ib, II, III, IV, V, VI, VII, VIII, and IX) was determined by multiplex polymerase chain reaction (PCR). Briefly, one colony of each isolate was transferred to a 10% Chelex-100 solution (Sigma-Aldrich, St. Louis, MO, USA), vortexed vigorously, and incubated in a dry bath at 95 °C for 30 min. The solution was then centrifuged at 4000 RPM for 30 s, and the supernatant was transferred to a microtube. Amplifications were performed in a total volume of 25 µL, in a MiniAmp thermal cycler (Applied Biosystems, Waltham, MA, USA), using five microliters of DNA in a multiplex PCR as previously described [15]. Additionally, the eight isolates selected for WGS (636, 659, 676, 690, 765, 782, 790, and 986) had their capsular types confirmed using the Group B Streptococcus (GBS) Typer pipeline (https://github.com/sanger-bentley-group/GBS-Typer-sanger-nf) accessed on 7 May 2024 [16].

### 2.4. Analysis of Clonality

Macrorestriction of chromosomal DNA using the SmaI enzyme (New England Biolabs, Ipswich, MA, USA), followed by pulsed-field gel electrophoresis (PFGE) was performed as previously described [6]. The run conditions were a 6 V/cm gradient, 23 h, 5 s initial switch time, and 35 s final switch time, using the CHEF Mapper system (Bio-Rad, Hercules, CA, USA). The gels were dyed with SYBR Safe DNA Gel Stain (Thermo Fisher Scientific, Waltham, MA, USA) and revealed using the ChemiDoc MP Imaging System (Bio-Rad, Hercules, CA, USA). The PFGE gel image was analyzed using Bionumerics software v. 7.6 (Applied Maths, Sint-Martens-Latem, Belgium) with 1.25% tolerance and 0.5% optimization.

### 2.5. Detection of Resistance and Virulence Genes

The ARGs (*ermA/TR*, *ermB*, *mefA*, *tetM*, and *tetO* genes) and virulence genes (*cylB*, *fbsB*, and *scpB* genes) were detected by PCR in all isolates. The DNA extraction was carried out as previously described for capsular serotyping. All amplifications were performed in a total volume of 20 µL, using a MiniAmp thermal cycler (Applied Biosystems, Waltham, MA, USA). Two microliters of DNA were added to a PCR reaction mix containing 0.20 µM of each primer (Table 1), 0.2 mM of each dNTP, 1 U Taq polymerase, 2 mM MgCl2, and reaction buffer 1X. The cycling parameters were as follows: 95 °C for 1 min, 35 cycles of 95 °C for 1 min, annealing for 1 min, extension at 72 °C for 1 min, and a final extension at 72 °C for 5 min (Table 1). The PCR product was run on a 2% agarose gel in Tris-acetate-EDTA buffer, stained with a UV nucleic acid stain (Sinapse Inc., São Paulo, Brazil), and visualized under a UV transilluminator. The results were evaluated based on the expected amplicon size (Table 1).

### 2.6. WGS and Bioinformatic Analysis

Eight isolates, selected to represent distinct clades identified in the clonality analysis, were randomly chosen for WGS. Sequencing was performed using a combination of short- and long-read technologies, following the methodologies previously described [6]. The obtained reads were assembled using Flye v.2.9.1 [20], with one polishing iteration, and hybrid assemblies were prepared using Polypolish v.0.5.0 [21]. Alternatively, the filtered long reads and trimmed short reads were assembled using Unicycler v.0.5.0 [22] and samtools v.1.15.1 [23], respectively, to compare the final assemblies. Graphical fragment assembly files were loaded into Bandage v.0.8.1 [24], and MLST v.2.22.0 [25] was used to determine sequence types (STs). The genomes were analyzed using the CGE service (https://www.genomicepidemiology.org/services) accessed on 7 May 2024, and mass screening of contigs for ARGs was performed using ABRicate v.1.0.19 (https://github.com/tseemann/abricate) accessed on 7 May 2024, and CARD RGI database v.3.2.7 [26]. BLASTn (https://blast.ncbi.nlm.nih.gov) was performed to identify the main virulence genes of *S. agalactiae*, and the pilus-coding genomic islands (PI-1A, PI-2A, and PI-2B) were determined using the GBS Typer pipeline [16] and manually checked in the hybrid-assembled genomes. The *S. agalactiae* Pathogenicity Islands (SagPAI) prediction was performed using GIPSy v.1.1.2 [27], and the results were manually curated according to Lannes-Costa et al. (2020) [28].

The sequenced genomes were assembled and annotated for phylogenomic analysis using Prokka v. 1.14.6 [29], and compared with Roary v. 3.13.0 [30]. The reference genomes NGBS128, 2603V/R, BM110, and MA12 (isolates from human hosts), and the 28 reference genomes from cow milk in Brazil, retrieved from the National Center for Biotechnology Information database (NCBI) on 5 December 2025 (Supplementary File S1), were annotated and loaded for comparison with this study’s isolates. A core genome phylogenetic tree was generated using IQ-TREE v2.0.7 [31] to infer the maximum likelihood and was visualized using FigTree v1.4.4 (https://tree.bio.ed.ac.uk/software/figtree) accessed on 30 November 2025.

## 3. Results

### 3.1. Bacterial Identification

In this study, a total of 91 bacterial isolates were obtained from bulk tank milk samples collected from four dairy farms located in the municipalities of Areia, Barra de Santana, Pilões, and Soledade, in the state of Paraíba, Brazil. Among these, 64 isolates showed typical colony morphology on blood agar, negative catalase and PYR test results, and a positive CAMP reaction. Species-level identification was confirmed by MALDI-TOF, which identified all 64 isolates as *S. agalactiae*, with confidence scores ranging from 1.80 to 2.41. Of these, 46 isolates were successfully recovered and preserved throughout all experimental procedures and were therefore included in the subsequent analyses.

### 3.2. Capsular Serotyping and PFGE Analysis

**Figure 2:**
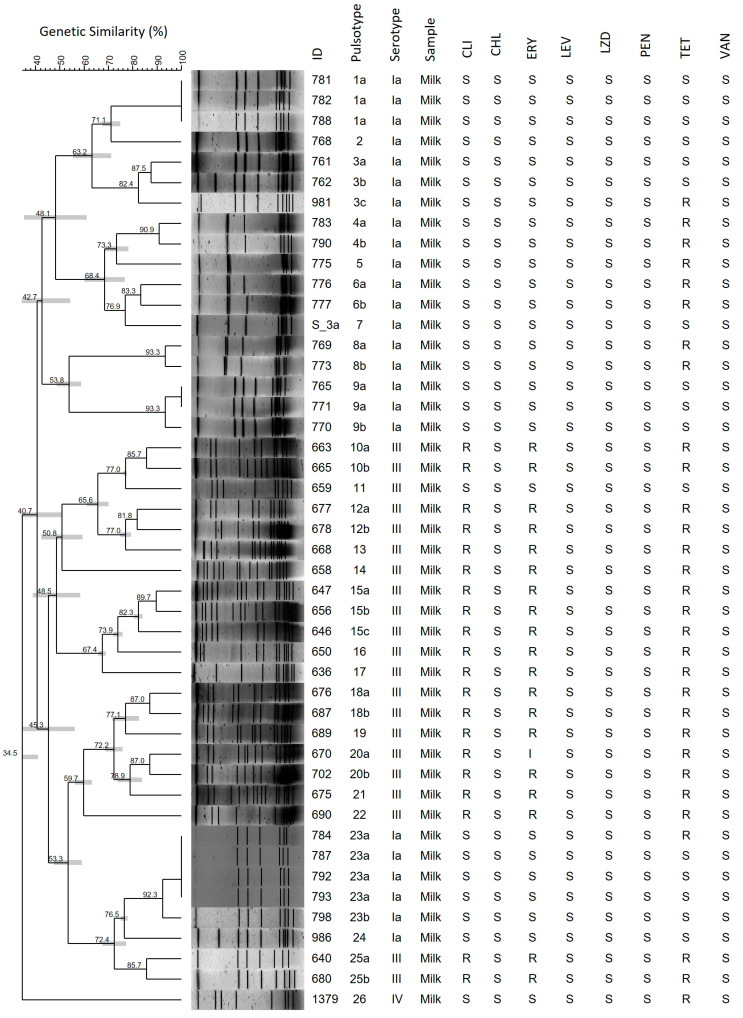
Dendrogram showing genetic similarity among 46 *S. agalactiae* isolates from dairy farms in Paraiba, Brazil, between 2018 and 2022, generated using Bionumerics software v. 7. 1 (Applied Maths, Belgium). A dendrogram was generated using 1.25% tolerance and 0.5% optimization. CLI: clindamycin; CHL: chloramphenicol; ERY: erythromycin; LEV: levofloxacin; LZD: linezolid; PEN: penicillin; TET: tetracycline; VAN: vancomycin; S: susceptible; I: intermediate; R: resistant.

Of the 46 isolates included in this study, the most prevalent serotype was Ia (n = 24/46, 52.2%), followed by III (n = 21/46, 45.6%), and IV (n = 1/46, 2.2%). Macrorestriction of chromosomal DNA, followed by PFGE, clustered the isolates into 26 pulsotypes (Figure 2).

### 3.3. Antimicrobial Susceptibility and Detection of ARGs by PCR

All the isolates were susceptible to CHL, LEV, LZD, PEN, and VAN. The PEN MIC values ranged from 0.032 to 0.094 µg/mL, with MIC_50_ and MIC_90_ of 0.047 and 0.094 µg/mL, respectively. The highest resistance index was found for TET (n = 30/46, 65.2%), followed by CLI (n = 20/46, 43.5%), and ERY (n = 19/46, 41.3%) (Figure 2); one isolate was intermediate for ERY. CLI-resistant isolates showed a constitutive MLSb (resistance to macrolides, lincosamides, and streptogramin B) phenotype. The *ermB* (n = 20/46, 43.5%) and *tetO* (n = 20/46, 43.5%) genes were the most prevalent determinants of resistance, and the *tetM* gene was also reported (n = 8/46, 17.4%). None of the isolates harbored the *ermA/TR* or *mefA* genes (Table 2).

### 3.4. Detection of Virulence Genes by PCR

PCR detection of virulence genes revealed a high prevalence of *cylB* and *fbsB* among the *S. agalactiae* isolates. All isolates analyzed (n = 46/46, 100%) were positive for both genes (*cylB* and *fbsB*). In contrast, the *scpB* gene was detected in only a single isolate (isolate 1379), corresponding to a prevalence of 2.2% (Table 2).

### 3.5. Genomic Characterization of Representative *S. agalactiae* Isolates

Eight of the 46 isolates were selected for WGS based on their representation of distinct clades identified in the clonality analysis. These included four serotype Ia isolates (782, 790, 765, and 986), corresponding to pulsotypes 1a, 4b, 9a, and 24, and four serotype III isolates (659, 636, 676, and 690), corresponding to pulsotypes 11, 17, 18a, and 22, respectively.

Among the eight sequenced isolates, four were assigned to ST-103 (n = 4/8, 50%), three to ST-91 (n = 3/8, 37.5%), and one was a single-locus variant (SLV) of ST-91 (n = 1/8, 12.5%), characterized by a non-typeable *glcK* allele (Table 3).

**Figure 3:**
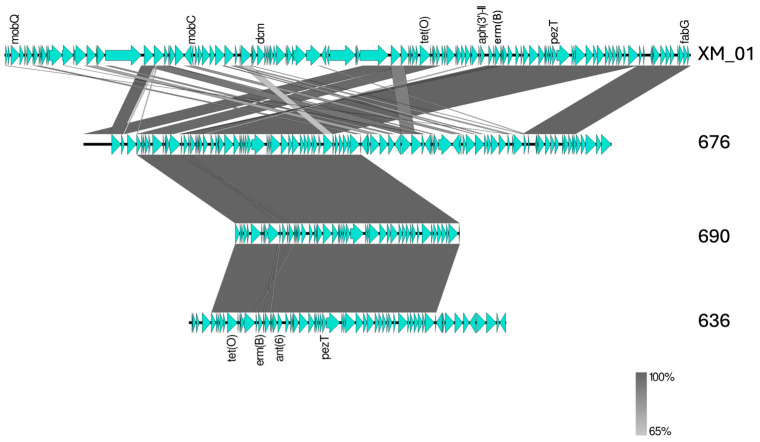
BLAST alignment of a genome region of three *S. agalactiae* isolates obtained from bovine raw milk in Paraíba, Brazil, between 2018 and 2022, and isolate XM_1 (accession number CP147645) from China. The aligned regions correspond to positions from 370,000 to 500,000 of isolate XM_1, contig 4 of isolate 676, contig 79 of isolate 636, and contig 9 of isolate 690. Arrows represent predicted coding sequences (genes), with their orientation indicating the direction of transcription. Gray blocks indicate regions of nucleotide similarity identified by BLASTn, with color intensity reflecting the degree of sequence identity. Comparative analysis was performed using BLASTn (https://blast.ncbi.nlm.nih.gov) and Easyfig version 2.2.

Two isolates belonging to ST-91 and the SLV of ST-91 harbored the ARGs *ant6-Ia*, *ermB*, and *tetO* within a 60 kb genomic region exhibiting high homology to the clinical *S. agalactiae* isolate XM_1 (accession number CP147645). In isolate 676, contig 4 comprised 230 kb and showed 99% nucleotide identity with XM_1, with 88% genome coverage. Only isolate 676 contained a complete *cyl* operon (*cylFEBAZCGDX*), whereas isolates 690 and 636 displayed transposase and insertion sequence elements upstream of the *ant6-Ia*, *ermB*, and *tetO* genes. This contig also harbored genes encoding a plasmid relaxosome protein (MobC), a relaxase-domain-containing protein, several phage-related proteins, and the replication initiation protein RepA (Figure 3). Among the ST-103 isolates, only one carried an antimicrobial resistance gene (*tetM*).

The virulence profile was homogeneous according to STs. All isolates contained PI-2B, *bca*, *cfb*, *cspA*, *cylE*, *fbsA*, *fbsB*, *fbsC*, *hylB*, *rib*, *sip*, and *pbsP*. Only isolates of ST-103 carried the *srr1* gene, and no sequenced isolate showed *bac*, *scpB*, *lmb*, or *hvgA* genes (Table 3).

**Figure 4:**
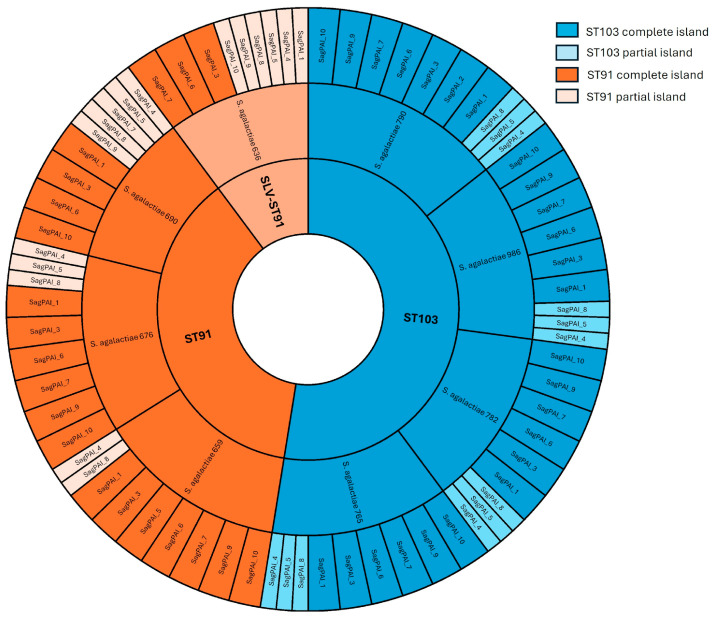
Comparative analysis of the presence of the 11 SagPAIs described in the hypervirulent *S. agalactiae* ST-17 lineage among eight representative *S. agalactiae* isolates obtained from dairy farms in Paraíba, Brazil, between 2018 and 2022.

The presence of complete or partial SagPAIs was investigated in the eight sequenced isolates by comparing their genomic profiles with those of the hypervirulent *S. agalactiae* ST-17 lineage, which is known to harbor 11 SagPAIs. SagPAI-11, which includes the *scpB* and *lmb* genes, was absent in all analyzed genomes. As shown in Figure 4, complete and partial SagPAIs were identified among the sequenced isolates. SagPAI-4 and SagPAI-8 were partially present in all isolates. Only isolate 659 harbored a complete SagPAI-5, whereas the remaining isolates contained this island only partially. SagPAI-2, which carries the *Tn916* element and the *tetM* gene, was detected exclusively in isolate 790.

**Figure 5:**
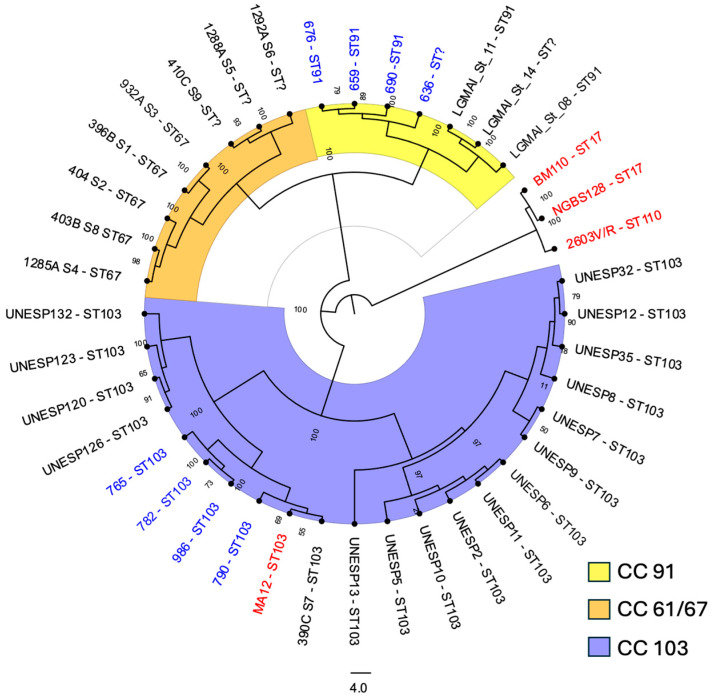
Genome phylogeny of *S. agalactiae* isolates from bovine milk in Brazil and reference strains retrieved from the NCBI GenBank database. Blue labels indicate the isolates sequenced in this study, obtained from dairy herds in Paraíba between 2018 and 2022. Black labels represent reference sequences from bovine milk in Brazil retrieved from the NCBI database, whereas red labels represent reference sequences obtained from human hosts and retrieved from the NCBI database. Node values indicate bootstrap support. CC: clonal complex.

A maximum-likelihood phylogenetic tree was constructed using the eight isolates sequenced in this study together with genome sequences retrieved from the NCBI GenBank database (Figure 5). Core genome phylogenetic analysis revealed three major lineages among *S. agalactiae* isolates obtained from bovine milk in Brazil, with ST-103 representing the predominant lineage, followed by ST-91 and ST-67. These lineages were genetically distant from human-adapted pathogenic lineages, such as ST-17. The four ST-103 isolates from this study (765, 782, 790, and 986) clustered with previously reported ST-103 isolates from the same region, including one isolate obtained from a human host in 2021 (MA12) and one isolate recovered from bovine milk in 2021 (390C S7). The three ST-91 isolates and the SLV-ST91 isolate clustered with isolates from Minas Gerais (LGMAI_St_11 and LGMAI_St_14) and São Paulo (LGMAI_St_8) states.

## 4. Discussion

Bovine mastitis is primarily of infectious origin and often caused by *S. agalactiae*, resulting in substantial losses in productivity and milk quality [5]. Additionally, milk can serve as a reservoir for resistant pathogens and mobile genetic elements carrying resistance genes, which can be transmitted to humans and other animals [32].

This study describes the genetic profiles of 46 *S. agalactiae* isolates from dairy farms in northeastern Brazil, contributing to improved mastitis control programs and milk quality monitoring. Genetic diversity analysis of the four herds showed that among the 21 isolates from Herd A, 14 pulsotypes were identified, all serotype III, with high resistance rates to CLI, ERY, and TET (95.2%). In Herd B, 10 pulsotypes were observed among 23 serotype Ia isolates, of which nine (39%) exhibited resistance only to TET. The remaining two pulsotypes corresponded to one serotype IV isolate from Herd C, which was also resistant to TET, and one serotype Ia isolate from Herd D, which was susceptible to all tested antibiotics. *S. agalactiae* is a highly contagious pathogen that is well-adapted to the udder environment, enabling its rapid spread within herds and causing both clinical and subclinical mastitis. Furthermore, during outbreaks, environmental transmission cycles may occur, posing significant challenges for the effective eradication of specific strains in dairy farms [5]. Thus, we observed high genetic diversity but phenotypic homogeneity within each herd, suggesting the persistence and local dissemination of specific strains within the herds.

Capsular polysaccharides are the major virulence factors of *S. agalactiae*, with ten recognized serotypes (Ia, Ib, II, III, IV, V, VI, VII, VIII, and IX). A multicenter study reported serotype III as the most prevalent serotype in Brazilian dairy farms; however, the distribution of serotypes varies regionally, with serotypes II, Ia, and III being the most common in northeastern herds [33]. In Argentina, a recent study identified serotypes III, II, and Ia, each predominating on individual dairy farms [34]. In our study, the most prevalent serotypes were Ia (52.2%) and III (45.6%), followed by IV (2.2%), reinforcing the association between serotype distribution and geographical region.

Surveillance of antimicrobial resistance in *S. agalactiae* is essential for effective mastitis control programs. Beta-lactams are first-line agents for *Streptococcus* infections, and reduced susceptibility of *S. agalactiae* isolates from bovine milk has emerged as a concern [12]. Isolates with PEN MIC ≤ 0.12 μg/mL are considered susceptible; in our study, the PEN MIC_90_ was 0.094 μg/mL, indicating that beta-lactams remain effective against *S. agalactiae* in bovine milk in northeastern Brazil.

Despite the high effectiveness of beta-lactams, resistance to macrolides and lincosamides such as ERY and CLI has increased over the last decade, coinciding with their expanded use. A meta-analysis revealed substantial geographic variation in ERY resistance, with the lowest prevalence in the Americas (14.9%) and the highest in Asia (43.4%) [10]. Recent studies of pregnant women in Brazil have reported an increasing prevalence of ERY resistance in the community (from 11.4% to 42.1%) after the COVID-19 pandemic, mainly driven by the *mefA* gene and less frequently by *ermA* and *ermB* [35,36], whereas resistance to CLI remained stable. In our study, we observed high resistance rates to ERY (41.3%) and CLI (43.5%) associated with the *ermB* gene, which encodes a 23S rRNA methyltransferase that confers resistance to macrolides, lincosamides, and streptogramin B. Indeed, *S. agalactiae* isolates from bovine origin in Brazil have previously been associated with higher ERY resistance rates (27.6–29.1%) [37,38]. In Brazil, combinations of macrolides and aminoglycosides are among the most frequently used intramammary therapies for mastitis [9], and their widespread use has contributed to increasing resistance levels. It is important to highlight that CLI is a key second-line therapy [10], particularly in human medicine, and the dissemination of *S. agalactiae* strains harboring *erm* genes raises concerns regarding their future reliability.

Excessive use of TET as a prophylactic agent or growth promoter in the past has led to high levels of resistance in *S. agalactiae*, primarily driven by ribosomal protection genes such as *tetM* and *tetO* [38]. The *tetM* gene, which is typically associated with the *Tn916* transposon family, is a characteristic feature of human-adapted strains and is less frequently found in bovine mastitis isolates. In contrast, *tetO* is the most common determinant in bovine strains [6,34]. In our study, TET resistance was the most frequent antibiotic-resistant phenotype. Among the resistant isolates, *tetO* was the predominant determinant, accounting for two-thirds of TET-resistant isolates, whereas *tetM* was detected in 26.7% of isolates. Furthermore, two isolates did not exhibit any of the tested resistance mechanisms, suggesting that other genotypes, such as *tetS*, *tetL*, or *tetT*, may be related to TET resistance in our setting [11].

Regarding ARGs, the combination of *ermB* and *tetO* was the most frequent genotype (43.5%) detected exclusively in serotype III isolates from a single herd (municipality A). A high prevalence of *tetO* and *ermB* has also been reported in Argentine herds, possibly associated with horizontally transferred genetic elements [34]. To further investigate this issue, we examined the genetic context of ARGs in three of these isolates (636, 676, and 690), identified as ST-91 or SLV of ST-91, and detected a genomic region with high similarity to that of an invasive *S. agalactiae* strain from the clonal complex (CC) 17 isolated in China (Figure 3). Moreover, the presence of *tetO* and *ermB* within a variant of the integrative conjugative element ICESag37 has been reported as a characteristic feature of two Brazilian clusters of ST-103 bovine strains [39]. These findings suggest that ARGs are integrated into the chromosomes of these isolates, supporting the possibility of the horizontal transfer of these resistance determinants among distinct *S. agalactiae* lineages.

Fibrinogen-binding proteins promote adherence to epithelial cells and are encoded by the *fbsA/B/C* gene family; *fbsB* is also associated with host cell invasion [8]. The cytolysin pigment, involved in cell invasion, tissue damage, and antioxidant activity, is encoded by the *cyl* operon, which comprises 12 genes, including *cylB* and *cylE* [40]. The *scpB* gene encodes C5a peptidase, an enzyme that inactivates the human complement factor C5a and plays a key role in adhesion and invasion in human-associated strains; however, it is less frequently observed in bovine isolates [8,40]. Regarding the prevalence of virulence genes, PCR detection of *cylB*, *fbsB*, and *scpB* demonstrated a homogeneous profile among isolates from the Paraíba herds. Only one isolate (2.2%) harbored *scpB*. For comparison, a study in Poland reported a prevalence of 35.3% [8], whereas no *scpB* was detected among ST-103 strains in Brazil [39]. The low prevalence of the *scpB* gene observed here suggests that *S. agalactiae* circulating in Paraíba herds may pose a reduced risk of invasive infections following transmission to humans.

Among the eight sequenced isolates, a broad repertoire of virulence factors was identified. These include *srr*, which is associated with epithelial cell adhesion; *cfb* and *bca*, which are related to tissue and cell injury; and *pbsP* and *hylB*, which are implicated in tissue invasion. Additional genes, such as *cspA*, *hylB*, *sip*, *rib*, and *bac,* have been identified and are known to interfere with host immune response mechanisms. Pili expression, which is an important determinant of persistent colonization and biofilm formation, was also observed in this study. *S. agalactiae* carries two pilus island variants (PI-1 and PI-2), and the PI-2B variant is considered characteristic of bovine-adapted strains [6]. Overall, the virulence gene profiles were largely homogeneous, differing only in the presence of *srr1*, which was exclusively detected in ST-103 isolates. As previously reported, *srr1* is a common feature of ST-103 strains obtained from both bovine and human hosts [39].

The SagPAI profiles revealed a reduced repertoire in ST-91 isolates compared with ST-103 isolates. Furthermore, our findings are consistent with those of previous studies that reported low conservation of several SagPAIs (2, 4, 5, and 8) across sequenced genomes. Additionally, we observed the absence of SagPAI-11, which contains *scpB* and *lmb* genes that are strongly associated with human-invasive isolates rather than bovine strains [6,28].

Phylogenetic analysis of the eight sequenced isolates from this study, together with genomes retrieved from the NCBI database, revealed the evolutionary relationships of *S. agalactiae* from bovine milk in Brazil, highlighting the clear evolutionary separation between human-specialized (CC17) and bovine-specialized lineages. The STs identified in this study (ST-91 and ST-103) have been previously reported in Brazilian dairy herds [33]. In the phylogenetic tree, ST-91 and ST-67 appeared to be most closely related, likely sharing a genetic background relevant to host adaptation, in contrast to ST-103. Indeed, clonal complex CC61/67 has never been reported in human hosts and is considered specialized for colonization of the bovine mammary gland, possibly driven by pseudogenization of the *cps* operon, which encodes the polysaccharide capsule; these CCs have been nearly eliminated from European herds [4]. In contrast, ST-103 was the most frequent lineage among the isolates in this study and across the NCBI-retrieved genomes. ST-103 is an important host generalist lineage in several countries, including Brazil [4,39]. Notably, one genome sequence (MA12), previously obtained from a human patient in Paraíba, Brazil [6], clustered with ST-103 isolates from this study. This finding underscores the potential for the inter-host transmission of ST-103.

Despite the relevance of these findings, some limitations must be considered when interpreting our conclusions. The isolates were obtained from only four municipalities in Paraíba, northeastern Brazil; therefore, the results cannot be extrapolated to other regions or production systems. The limited number of sequenced isolates also restricts their ability to fully capture existing genomic and population variability. In addition, the study did not include data on farm management practices, interventions, or antibiotic use, which could have influenced the introduction, persistence, and adaptation of specific lineages. Finally, further studies with broader geographic coverage and expanded sampling are necessary to confirm and refine these observations across the different regions of Brazil.

## 5. Conclusions

Forty-six *S. agalactiae* isolates obtained from bulk tanks in northeastern Brazil were investigated. PFGE revealed high overall genetic diversity; however, virulence and resistance profiles were largely homogeneous within each herd, reinforcing the contagious nature of *S. agalactiae* and its local dissemination pattern. Antimicrobial resistance was common and was primarily driven by *tetO* and *ermB* genes. WGS demonstrated that these resistance determinants were integrated into the mobile genetic elements found in human clinical isolates. Additionally, ST-103 was the most prevalent lineage, and phylogenetic analyses highlighted its multihost-adapted status, whereas ST-91 clustered with the bovine-adapted CC61/67 complex. Together, these findings expand the molecular epidemiological understanding of *S. agalactiae* on northeastern Brazilian dairy farms and support the development of improved mastitis control strategies and milk quality management practices.

## Figures and Tables

**Table 1:** Oligonucleotide primer sequences for PCR assays to detect resistance and virulence genes in *S. agalactiae* from dairy farms in Paraiba, Brazil, between 2018 and 2022.

Target	Sequence (5′–3′)	Size (bp)	Annealing Temperature (°C)
*cylB* [17]	GGGCTGCAGGTATTATCGAA ATTTCCACCAAAAGCAAACG	176	60
*ermA/TR* [18]	AACTTGTGGAAATGAGTCAACGG CAGAATCTACATTAGGCTTAGGG	375	60
*ermB* [18]	ATTGGAACAGGTAAAGGGCG GAACATCTGTGGTATGGCG	442	60
*fbsB* [17]	ACAACTGCGGAAATGACCTC ACGAGCGACGTTGAATTCTT	186	60
*mefA* [18]	AGTATCATTAATCACTAGTGC TTCTTCTGGTACTAAAAGTGG	345	50
*tetM* [19]	GTGGAGTACTACATTTACGAG GAAGCGGATCACTATCTGAG	359	50
*tetO* [19]	GCGGAACATTGCATTTGAGGG CTCTATGGACAACCCGACAGAAG	538	50
*scpB* [17]	AGCCATATGCTGCGATCTCT GGGTTGAACCAAGTGTGCTT	198	60

**Table 2:** Molecular diversity, capsular type, virulence, and resistance genes of 46 *S. agalactiae* from dairy farms in Paraiba, Brazil, between 2018 and 2022.

Municipalities	Isolates (n)	Pulsotype	Capsular Type	Virulence Genes	Resistance Genes
A	3	15	III	*cylB*, *fbsB*	*ermB*, *tetO*
A	2	10	III	*cylB*, *fbsB*	*ermB*, *tetO*
A	2	12	III	*cylB*, *fbsB*	*ermB*, *tetO*
A	2	18	III	*cylB*, *fbsB*	*ermB*, *tetO*
A	2	20	III	*cylB*, *fbsB*	*ermB*, *tetO*
A	2	25	III	*cylB*, *fbsB*	*ermB*, *tetO*
A	1	11	III	*cylB*, *fbsB*	*-*
A	1	13	III	*cylB*, *fbsB*	*ermB*, *tetO*
A	1	14	III	*cylB*, *fbsB*	*ermB*, *tetO*
A	1	16	III	*cylB*, *fbsB*	*ermB*, *tetO*
A	1	17	III	*cylB*, *fbsB*	*ermB. tetO*
A	1	19	III	*cylB*, *fbsB*	*ermB*, *tetO*
A	1	21	III	*cylB*, *fbsB*	*ermB*, *tetO*
A	1	22	III	*cylB*, *fbsB*	*ermB*, *tetO*
B	5	23	Ia	*cylB*, *fbsB*	*-*
B	3	1	Ia	*cylB*, *fbsB*	*-*
B	3	3	Ia	*cylB*, *fbsB*	*-*
B	3	9	Ia	*cylB*, *fbsB*	*-*
B	2	4	Ia	*cylB*, *fbsB*	*tetM*
B	2	6	Ia	*cylB*, *fbsB*	*tetM*
B	2	8	Ia	*cylB*, *fbsB*	*tetM*
B	1	2	Ia	*cylB*, *fbsB*	*-*
B	1	5	Ia	*cylB*, *fbsB*	*tetM*
B	1	24	Ia	*cylB*, *fbsB*	*-*
C	1	26	IV	*cylB*, *fbsB*, *scpB*	*tetM*
D	1	7	Ia	*cylB*, *fbsB*	*-*

A: Areia; B: Barra de Santana; C: Pilões; D: Soledade; *-*: not detected.

**Table 3:** Epidemiological, resistome, and virulome features of eight representative isolates of *S. agalactiae* from dairy farms in Paraiba, Brazil, between 2018 and 2022.

Isolate	Sequence Type	ARGs	Virulence Genes	Pilus Island	Isolates Accession Number
636	SLV 91	*ant6-Ia*, *ermB*, *tetO*	*bca cfb cspA cylE fbsA fbsB fbsC hylB rib sip pbsP*	2B	JBCPXS000000000.1
659	91	*-*	*bca cfb cspA cylE fbsA fbsB fbsC hylB rib sip pbsP*	2B	JBBEFD000000000.1
676	91	*ant6-Ia*, *ermB*, *tetO*	*bca cfb cspA cylE fbsA fbsB fbsC hylB rib sip pbsP*	2B	JBANBL000000000.1
690	91	*ant6-Ia*, *ermB*, *tetO*	*bca cfb cspA cylE fbsA fbsB fbsC hylB rib sip pbsP*	2B	JBANBM000000000.1
765	103	-	*bca cfb cspA cylE fbsA fbsB fbsC hylB rib sip pbsP srr1*	2B	CP143102.1
782	103	*-*	*bca cfb cspA cylE fbsA fbsB fbsC hylB rib sip pbsP srr1*	2B	CP142854.1
790	103	*tetM*	*bca cfb cspA cylE fbsA fbsB fbsC hylB rib sip pbsP srr1*	2B	JAYXIU000000000.1
986	103	-	*bca cfb cspA cylE fbsA fbsB fbsC hylB rib sip pbsP srr1*	2B	CP142853.1

ARGs: antibiotic resistance genes; SLV: single-locus variant; -: absent.

## Data Availability

The original contributions of this study are included in the article/supplementary materials. The assembly files and annotated genomes are available on the NCBI database (accession numbers available on Supplementary File S2). Raw sequencing data (Illumina and Nanopore files) and further inquiries can be directed to the corresponding author.

## Supplementary Materials

The following supporting information can be downloaded from https://www.mdpi.com/article/10.3390/pathogens15020128/s1. File S1: List of reference strains retrieved from the NCBI database; File S2: General information and accession numbers of sequenced isolates; File S3: Pathogenicity islands report in the sequenced isolates.

## Abbreviations

The following abbreviations are used in this manuscript:

ARG	Antibiotic resistance gene
CC	Clonal complex
CHL	Chloramphenicol
CLI	Clindamycin
CLSI	Clinical Laboratory Standard Institute
ERY	Erythromycin
LEV	Levofloxacin
LZD	Linezolid
MGE	Mobile genetic element
MIC	Minimum inhibitory concentration
MLSb	Resistance to macrolides, lincosamides and streptogramin B
NCBI	National Center for Biotechnology Institute
PCR	Polymerase chain reaction
PEN	Penicillin
PFGE	Pulsed-field gel electrophoresis
PI	Pilus island
SLV	Single locus variant
ST	Sequence type
TET	Tetracycline
VAN	Vancomycin
WGS	Whole-genome sequencing
